# GRYFUN: A Web Application for GO Term Annotation Visualization and Analysis in Protein Sets

**DOI:** 10.1371/journal.pone.0119631

**Published:** 2015-03-20

**Authors:** Hugo P. Bastos, Lisete Sousa, Luka A. Clarke, Francisco M. Couto

**Affiliations:** 1 LaSIGE, Departamento de Informática, Faculdade de Ciências, Universidade de Lisboa, Lisboa, Portugal; 2 Departamento de Estatística e Investigação Operacional e Centro de Estatística e Aplicações, Faculdade de Ciências, Universidade de Lisboa, Lisboa, Portugal; 3 BioFIG - Centre for Biodiversity, Functional and Integrative Genomics, Faculdade de Ciências, Universidade de Lisboa, Lisboa, Portugal; 4 LaSIGE, Departamento de Informática, Faculdade de Ciências, Universidade de Lisboa, Lisboa, Portugal; Universita’ di Padova, ITALY

## Abstract

Functional context for biological sequence is provided in the form of annotations. However, within a group of similar sequences there can be annotation heterogeneity in terms of coverage and specificity. This in turn can introduce issues regarding the interpretation of actual functional similarity and overall functional coherence of such a group. One way to mitigate such issues is through the use of visualization and statistical techniques. Therefore, in order to help interpret this annotation heterogeneity we created a web application that generates Gene Ontology annotation graphs for protein sets and their associated statistics from simple frequencies to enrichment values and Information Content based metrics. The publicly accessible website http://xldb.di.fc.ul.pt/gryfun/ currently accepts lists of UniProt accession numbers in order to create user-defined protein sets for subsequent annotation visualization and statistical assessment. GRYFUN is a freely available web application that allows GO annotation visualization of protein sets and which can be used for annotation coherence and cohesiveness analysis and annotation extension assessments within under-annotated protein sets.

## Introduction

The functional annotation of biological sequences is a crucial step for their biological contextualization. Such annotations can be derived from biological experimentation or other kinds of evidence such as sequence similarity through expert curation. However, biological experimentation and curation are very time and resource consuming tasks and thus this kind of approach is unable to keep up with the current rate of biological sequencing. Therefore, most (>98%) of the existing functional annotations are assigned by automatic annotation methods [1]. Therefore, it is critical that these automatic methods achieve high accuracy. For this purpose, initiatives like the Critical Assessment of protein Function Annotation (CAFA) experiment are held to analyse and evaluate the current state-of-the-art protein function prediction methods and how they tackle different difficulties presented in protein prediction [2].

There are several issues and challenges to protein functional prediction and annotation [3] and among them is the fact that annotations are often incomplete or can even be erroneous. Furthermore, in the case of erroneous annotations this can be even more problematic for automatic methods which have a greater potential for error propagation and increased difficulty in backtracking such errors. Hence, the global result of all the annotation methods is an heterogeneous annotation landscape in terms of annotation quality, completeness and specificity.

The Gene Ontology (GO) Consortium aims at providing generic and consistent descriptions for the molecular phenomena in which the gene products are involved. Given their broad scope and wide applicability the GO aspects have become the most popular of ontologies for describing gene and protein biological roles. For that purpose the GO project provides three growing orthogonal ontologies, or aspects, that describe gene product phenomena at different levels: biological processes, cellular components and molecular functions [4]. Structurally, the terms in each GO aspect are organized as DAGs (Directed Acyclic Graphs) where each node represents a concept (term) and the edges represent a relationship between those concepts. Those relationships between concepts can be of three types: *is_a*, *part_of* and *regulates*.

A plethora of bioinformatics tools relying on GO have been developed either by the GO Consortium or by other third parties (http://neurolex.org/wiki/Category:Resource:Gene_Ontology_Tools). These tools can serve different purposes, depending on the research focus each tool was designed for. Among these tools, visualization and statistical analysis are commonly implemented features.

Perhaps, one of the most relevant contributions of Statistics to GO term studies, concerns the enrichment analysis. For instance, micro-array experiments often output lists which can represent hundreds or even thousands of genes that are found to be differentially regulated for a given condition under study. Hence, in this case, the purpose of term enrichment analysis is to identify a representative set of activity terms that summarize the particular biological activities that are characteristic of the particular condition under study. The actual decision as to whether enrichment (or depletion) of annotation terms occurs in any given set is done by resorting to commonly used test statistics such as the Fisher exact test and the Chi-squared test. Additionally, Huang, *et al*. [5] collected and reviewed 68 bioinformatic enrichment tools while categorizing them into three different classes: singular enrichment analysis (SEA), gene set enrichment analysis (GSEA) and modular enrichment analysis (MEA). For any of these classes, the decision whether the enrichment is relevant is made on the basis of the obtained p-values. For the SEA class methods the p-values are calculated for each term in a list of pre-selected genes deemed of interest, while for the GSEA class methods experimental values are directly integrated into the calculation of the p-values with no need for pre-selection. On the other hand, methods of the MEA class are similar to those of the SEA class, except they include term-term and gene-gene relations into their procedures.

When coupled with enrichment analysis, (graph) visualization of annotations can help with the analysis by enabling the visual identification of the existing relationships between annotation terms found to be enriched. Bioinformatic tools like GOBar [6], GOLEM [7], GOrilla [8], StRAnGER [9] among several other tools provide this combination of enrichment analysis and annotation visualization. That is, all of these tools generate and display graphs that subsume the annotation terms of a target gene (or protein) set in addition to computing the respective enrichment values. On the other hand, a tool such as PANADA [10] also provides visualization but instead of annotation centric visualizations it generates protein similarity networks.

Typical enrichment analysis methodologies, especially of the SEA and GSEA classes, do present several limitations [5], [11] chief among them being that the statistics used (considering, for example, test statistics following a hypergeometric distribution, chi-square distribution, etc., under the null hypothesis) do not depend on measured changes. Furthermore, the underlying distribution of genes among various functions may not be uniform, so typically inference regarding enrichment of annotation terms must be made with care [6]. The present paper introduces and discusses the **gr**aph anal**y**ser of **fun**ctional annotation (GRYFUN) web application. This application provides visualization and statistical metrics of functional annotations for input protein sets. GRYFUN, despite being similar to the aforementioned tools is in turn particularly designed and focused on the analysis of the functional annotations in protein families or in functionally related sets of proteins. That is, GRYFUN provides an organizing data schema that allows for the fitting of proteins into Super-families>families, such as they are commonly organized in many specialized protein databases. This can in turn enable the harnessing of knowledge implicit in such protein groupings. Furthermore, the most distinctive feature in GRYFUN is the ability to interactively create new annotation sub-graphs out of regions on a parent graph that are deemed of particular interest by the researcher and are supported by the associated enrichment statistics. Hence, a non-uniform annotation distribution, in this case, can in fact be used as an advantage in determining the functional focus of a given target protein family (or functionally related protein set).

Here we present GRYFUN, a web application focused on annotation analysis annotation coherence and cohesiveness assessments and that is also capable of providing potential annotation extension assistance for under-annotated protein sets.

## Implementation

### Tool Description

GRYFUN can be publicly accessed and is available as a web-based application at: http://xldb.di.fc.ul.pt/gryfun/

The main function of GRYFUN is to generate graphs subsuming the GO annotations of input protein sets. Just like GObar [6] and GOrilla [8] the graph generation output process is handled by the GraphViz [12, 13] visualization software package. The GOA project [14] provides a suitable body of GO annotations for GRYFUN. The underlying database is built from the sources of the UniProt knowledgebase February 2014 release as well as the corresponding UniProt gene association (GOA) tables and respective Gene Ontology (GO) tables. The implementation of this tool relies on the Python Web Framework web2py technology.

### Input

GRYFUN currently only accepts UniProt accession numbers as input protein identifiers in order to create a user protein Set. In turn, each Set must belong to a Collection which is just a group of Sets. Additionally, Collections while providing a way to group Sets that share some functional similarity, can also and consequently create a coarser level of granularity. However, a proper use of the Collection/Set organization is paramount in p-value calculation and subsequent GO term enrichment decisions. For any given Set, the statistical tests are applied to determine the statistical significance of any given annotation term in the Set being explored, with the remaining Sets in that Collection being the *background set*

Thus, the remainder of Collection should be constructed in order to contain the complementary proteins of the Set to be analysed. For instance, in the classic case of the analysis of a group of differentially expressed proteins from a micro-array experiment, a Collection would have at least two Sets, the *differentially expressed set* and the *micro-array set* containing all the proteins in the micro-array. On the other hand, the Set/Collection partitioning is perfect for inserting protein families, as Sets that belong to Super-families (Collections).

The input proteins in each Set are expected to have a close degree of functional similarity, such as is the case of functional protein families or other groups of functionally related proteins. Alternatively, a Set can host dissimilar proteins if the intended purpose is just to navigate the generated annotation graph and manually sort and select sub-sets of proteins.

### Graph Visualizations

**Fig 1:**
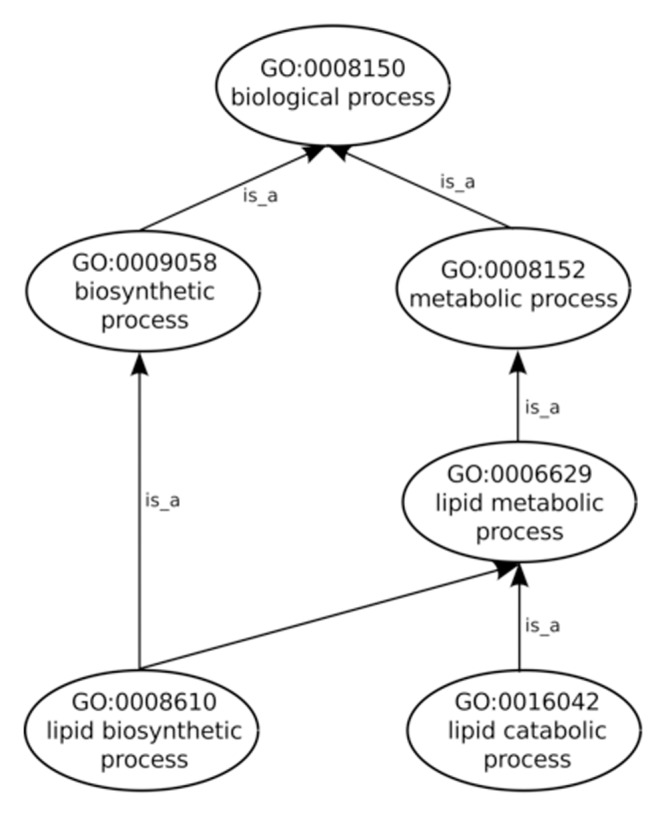
GO graph. Sub-graph of the GO *biological process* sub-ontology depicting *is_a* relationships.

After the input of protein Sets into their appropriate Collections the generation of annotation graphs is enabled. This is the central feature of GRYFUN and all the subsequent analysis is derived from these graphs and their supporting metrics and statistics. The annotation graphs generated by GRYFUN are very similar and dependent on GO graphs, however they present a couple of important differences. A GO graph is meant to denote relationships between terms, so each term is represented by a node whereas the relationships between terms are denoted by graph edges. Fig. 1 shows a GO sub-graph depicting nodes of the *biological_process* GO sub-ontology connected by *is_a* edges. Each of these edges starts at a child node (term) and points towards a parental node (term), and thus denotes the existing hierarchical relationship between terms. Additionally, all terms converge into a common root node, thus leading to the true path rule that states that “the pathway from a child term all the way up to its top-level parent(s) must always be true” [4].

**Fig 2:**
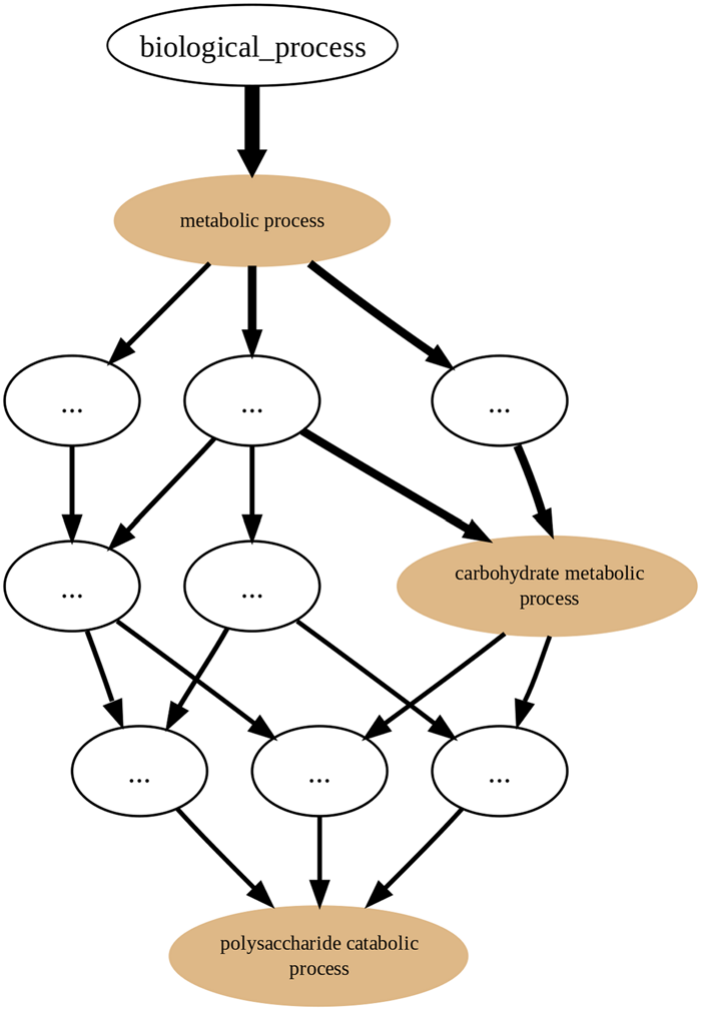
GRYFUN annotation graph. Example of a GRYFUN annotation graph subsuming the GO *biological process* sub-ontology annotations in a sample protein set.

On the other hand, in the GRYFUN annotation graphs, for example, the one shown in Fig. 2, the edge direction is reversed. Every protein in a Set generating an annotation graph is mandatorily annotated to at least the root term (*biological_process* in this case). Depending on how well-annotated any given protein is, it will “flow down” the graph towards more specific nodes. That “flow” can be immediately discernible from the annotation graph given that the edge thickness is proportional to the number of proteins that “flow down” from one parent node to its child node. In fact, what is happening in an annotation graph is that undirected edges between protein accessions and their respective GO annotation terms are being added to the original GO DAG nodes. Thus, proteins annotated to highly specific terms will be associated with a path of related nodes leading to one or more specific nodes. Therefore, by representing the “annotation flow” on the graph image, an immediate visual cue is provided regarding the annotation terms that are more frequent in any given protein Set and at the same time how they relate to each other. Furthermore, this type of annotation graph enables analyses such as the ones previously proposed by the authors [15].

### Term Enrichment

While the “annotation flow” in the annotation graph provides a visual cue of the more frequent terms in a given Set, term enrichment will allow users to ascertain the statistical significance of such terms. A commonly used *term-for-term* approach is applied in GRYFUN to determine enrichment of GO term annotations in protein Sets. For any given term annotation in a (study) Set, the purpose is to test the null hypothesis that states that there is no association between the number of annotated proteins in a Set and the number of annotations of that given term, against the alternative hypothesis of association between them. That is, each Set is considered to be, by the null hypothesis, just a random sample of the population, which in GRYFUN is defined as the Collection, hence the importance of defining relevant Collections when adding data. The statistical evidence of enrichment is postulated if the p-values are small, these p-values being calculated by the Fisher’s exact test. However, the graph nature of GO causes an issue with statistical dependencies when using the *term-for-term* approach, that is, for a given term annotating a certain number of proteins, at least that same number of proteins or more will also be annotated by the parental terms. Thus, in order to mitigate this propagation issue a Topology-based Elimination (Elim) strategy [16, 17] (using a significance level of 0.05) was implemented using the Python programming language. Given that the computed p-values for the GO terms under this strategy are conditioned on their children terms, and thus not independent, direct application of the multiple testing theory is not possible. It is then preferable to interpret the returned p-values as corrected or not affected by multiple testing.

### IC-based term score

A commonly used node property is the IC, which is a frequency-based measure of how specific a term is within a given corpus [18]. The IC of a term can then be given by the following equation:

$$\begin{array}{c} IC\left(t\right)=-log_{2}f\left(t\right) \end{array}$$ (1)

where *f(t)* is the fraction of proteins annotated to term *t* or any of its descendants in the annotation corpus. The GOA project [14] provides a suitable body of GO annotations and is used in GRYFUN as an annotation corpus. Furthermore, in GRYFUN the IC is scaled as previously described [19] using the following equation:

$$\begin{array}{c} IC_{u}\left(t\right)=\frac{IC(t)}{log_{2}N} \end{array}$$ (2)

where *N* is the total number of annotations within the corresponding GO sub-ontology being considered.

Finally the empirical IC-based term score is calculated through the use of the following equation:

$$\begin{array}{c} IC_{score}\left(t\right)=IC_{u}\left(t\right)\times s\left(t\right) \end{array}$$ (3)

where *s*(*t*) is the frequency of annotation of term *t* in the current Set in order to weight the cardinality of a given term with a metric of its relative specificity. The IC-based term score can then be used as a second tier of sorting among the significant terms revealed (through their respective p-values) by the term enrichment procedure.

### Graph Re-root

One of the most interesting features of GRYFUN is the ability to *re-root* annotation graphs. This feature is similar to the GOLEM [7] focus feature which reduces the graph to a selected GO annotation term and its vicinity (parents and children). On the other hand, GRYFUN’s *re-root* allows the selection of any non-leaf term node in an annotation graph followed by generation of a new sub-graph rooted at the term represented by the chosen node.

A typical good choice for a *re-rooting* operation would be a non-leaf node representing a term bearing a large IC-based term score that is also deemed significant by its p-value. Thus, performing a *re-rooting* operation will create a new annotation sub-graph subsuming only the annotations that are descendants of the new chosen root term, and thus considering only the proteins annotated with this new root term and its descendants, despite keeping a Set whole.

Hence, this feature enables the focus on more specific functional branches and terms of interest while abstracting from terms sometimes describing accessory activities that despite being associated to some proteins in a set can be considered as noise. The IC-based term score will remain the same after a *re-rooting* operation for the retained terms because this metric is only dependent on the frequency of the term itself both in the current Set and in the background GOA annotation corpus. On the other hand, the p-values obtained after a *re-rooting* procedure are subject to change because statistical testing is done over the *current graph view* and thus, does not consider the *masked* annotation terms.

### Graph Interactivity

The annotation graphs displayed by GRYFUN while apparently static do offer some interactive elements. The colored nodes represent the direct annotations while the white unlabelled notes the inherited ones. However, hovering the mouse cursor over any node will display a tooltip with the full term name plus its annotation frequency within that Set. Furthermore, clicking on a node will open a new floating modal window listing all the protein identifiers that are annotated to the term represented by the clicked node. The displayed protein list can be exported, either entirely or a selection, as a simple tab separated value (TSV) file for use with external programs and subsequent analysis. Additionally, the *re-rooting* feature is accessible from within these dynamically generated information floating modal windows (as well as directly from the term statistics information tables).

## Datasets

In order to illustrate the feasibility of GRYFUN we demonstrate its use with four different datasets.

### CAZy (Polysaccharide Lyases)

The CAZy database (http://www.cazy.org) describes the families of structurally-related catalytic and carbohydrate-binding modules (or functional domains) of enzymes that degrade, modify, or create glycosidic bonds [20]. Additionally, the classification into families and subfamilies in the CAZy database is based on amino acid sequence similarities, intended to reflect their structural features [21]. Furthermore, since CAZy is a curated knowledgebase of functionally related protein (module) families, and because it does not make use of GO as primary annotation system it presents itself as a good candidate on which to perform GO annotation coherence assessments and annotation extension studies using the GRYFUN web application. Hence, from this database we extracted protein identifiers to create a GRYFUN Collection comprising the families from the four CAZy catalytic module classes (n = 138676). Additionally, we created a Collection containing only the 21 families belonging to the Polysaccharide Lyase (PL) class (n = 1839). Each Set in those Collections matched the public UniProt protein primary entries (as of October, 2014) found in the CAZy website for each of the families in the CAZy database.

### YHTP2008

The CYC2008 project (http://wodaklab.org/cyc2008/) makes available two catalogues of yeast protein complexes resulting of systematic curation efforts. The first one, CYC2008, is a comprehensive catalogue of 408 manually curated heteromeric protein complexes reliably backed by small-scale experiments reported in the current literature [22]. Whereas the second catalogue, YHTP2008, comprises 400 high-throughput complexes derived from high-throughput Tandem Affinity Purification/Mass Spectrometry (TAP/MS) studies [23]. These are either manually annotated with current literature citations if they share subunits with literature-reported complexes, or marked as putative complexes if they are not yet characterized in any small-scale studies. We have downloaded this latter catalogue, and each cluster with four or more UniProt identifiers was recreated as a Set in a GRYFUN Collection totalling 133 Sets (n = 1255).

### MEROPS

The MEROPS database (http://merops.sanger.ac.uk) aims at providing an integrated source of information on peptidases, their substrates and inhibitors. This database has hierarchical classifications in which homologous sets of peptidases and protein inhibitors are grouped into protein species, which are grouped into families, which are in turn grouped into clans [24]. The family classification in this database also makes it convenient for functional annotation assessments using the web application GRYFUN. Thus, we extracted all the UniProt identifiers from this database (MEROPS release 9.9) family classifications. Families with four or more identifiers were recreated as Sets in a GRYFUN Collection that was finally composed of 238 Sets (n = 93124).

### Gene Expression Micro-array Data

We made use of a small-scale micro-array study of differential gene expression in human native nasal epithelial cells from five F508del-homozygous cystic fibrosis (CF) patients vs. five control individuals [25]. Data analysis using the Rank Products method [26] resulted in a list of differentially expressed genes, many of which were found to be functionally relevant to CF pathophysiology, based on GO term enrichment using (e.g.) the open-source software package DAVID [5]. For the GRYFUN analysis we converted genes up-regulated 2-fold or more in CF samples compared to controls into Uniprot accession IDs (n = 150), and ran this single set against a background of Uniprot IDs (n = 9083) converted from named genes on the Affymetrix HsAirway micro-array used in the study. The results obtained with GRYFUN were then compared with those obtained for the same gene list using two other GO term enrichment software platforms; GOrilla [8], using the same background as GRYFUN, and DAVID, using the default *H. sapiens* background.

## Results and Discussion

### Polysaccharide Lyases

**Fig 3:**
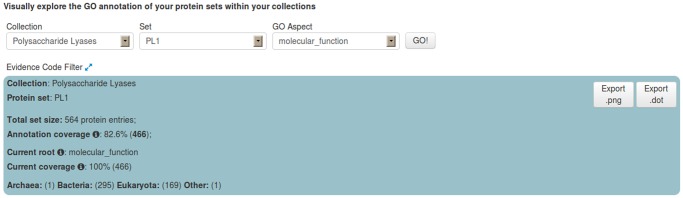
PL1 set summary header. Header with summary statistics regarding the PL1 (CAZy family) Set respective to the GO *molecular function* sub-ontology annotation coverage.

**Fig 4:**
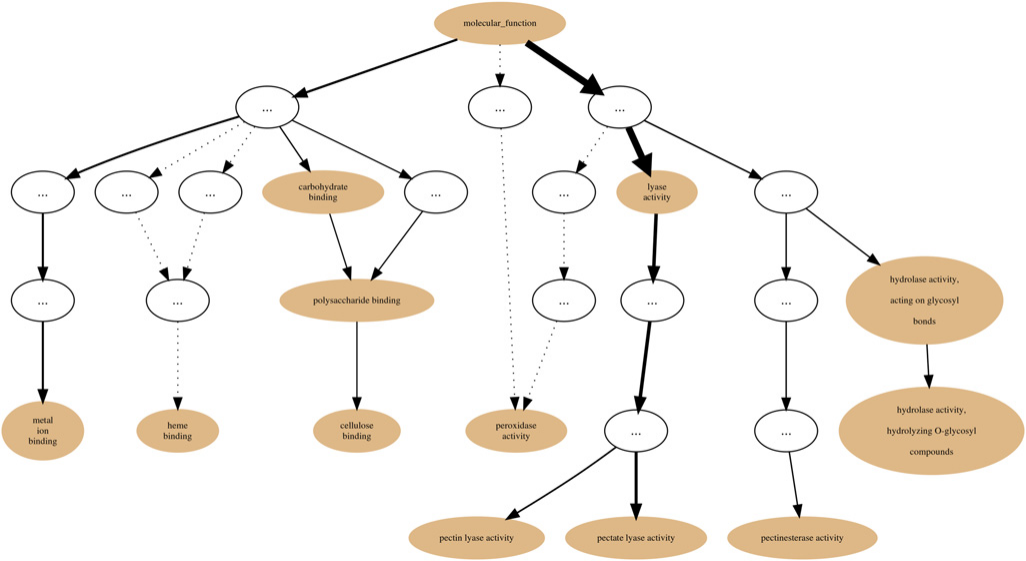
PL1 annotation graph. Annotation graph subsuming the PL1 (CAZy family) Set GO *molecular function* sub-ontology annotations.

**Fig 5:**
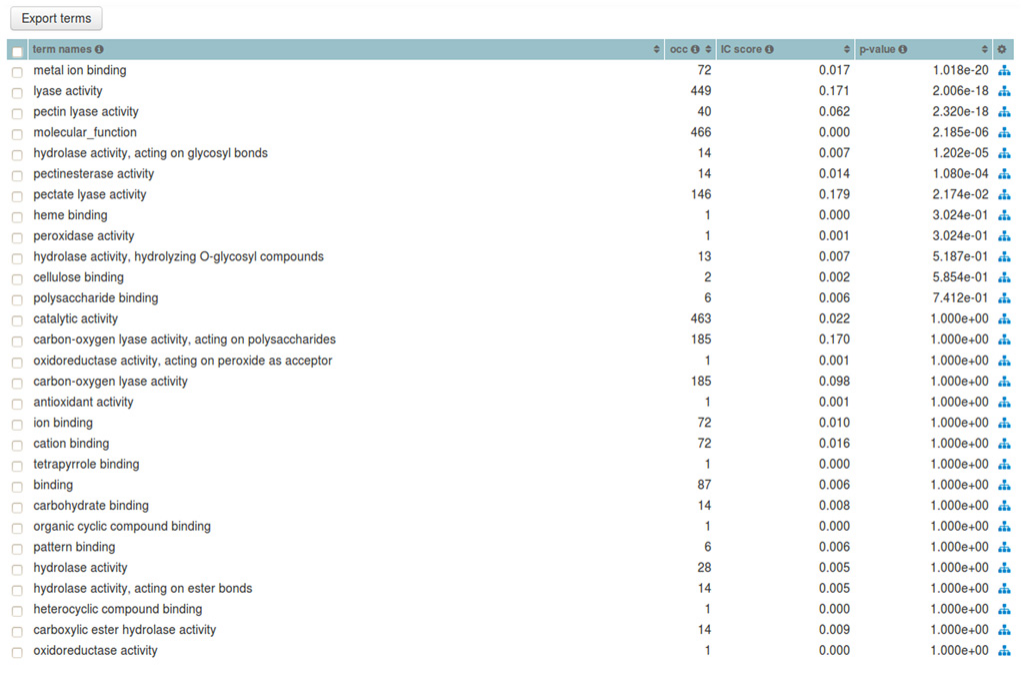
Term statistics for the PL1 Set. Footer table listing the *molecular function* GO term names and their respective statistics, such as, occurrence (occ), IC-based score and p-value for the PL1 (CAZy family) Set.

**Fig 6:**
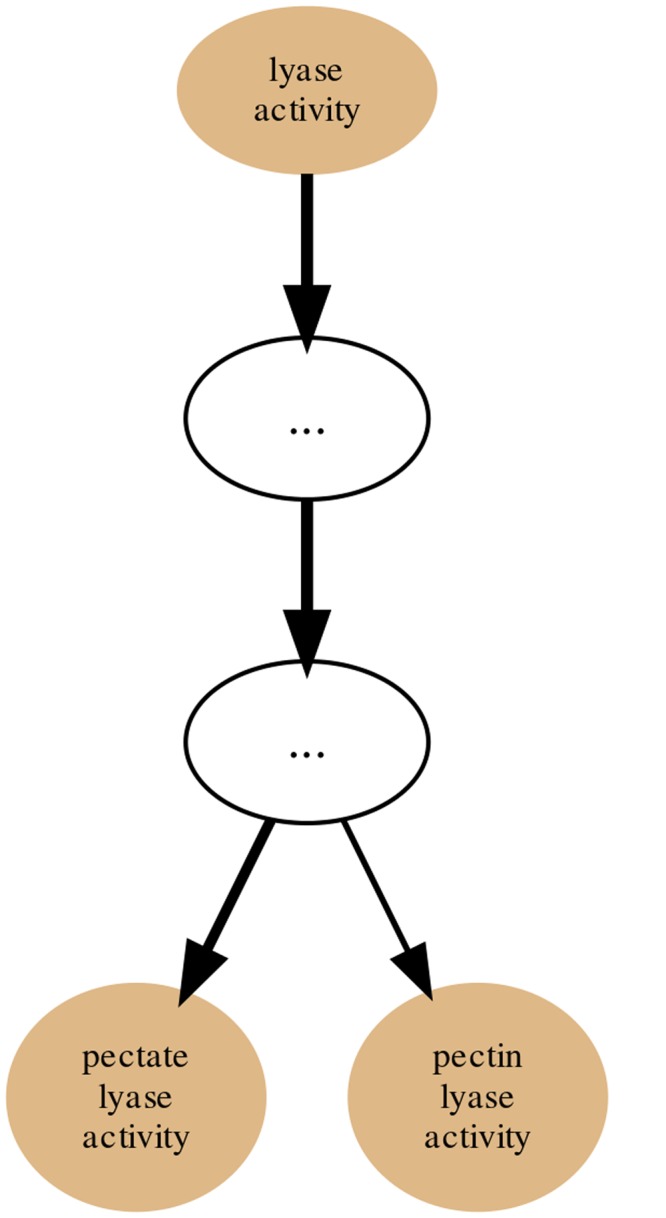
PL1 Set annotation graph *re-rooted* at the *lyase activity* term. Annotation graph of the PL1 (CAZy family) Set for the GO *molecular function* sub-ontology *re-rooted* at the *lyase activity* term.

Within the PL families dataset we chose, as an example, the PL1 Set (family) and generated its annotation graph and associated statistics for the *molecular function* sub-ontology of GO by using the GRYFUN web application. All evidence code annotation types were considered. The PL1 family/set is comprised of 564 UniProt protein entries of which 466 are annotated with terms from the GO *molecular function* sub-ontology. This information is also displayed at the header of the generated page as shown in Fig. 3. In addition, the header also displays information such as the Superkingdom taxonomical breakdown of the proteins in the current Set. The central element of the dynamically generated page is an interactive annotation graph such as the one depicted in Fig. 4 for the PL1 set. Visual inspection of the graph immediately makes evident that the main *annotation flow* occurs from the root term (*molecular function*) towards the two leaf-terms: *pectate lyase activity* and *pectin lyase activity*. Furthermore, by inspecting the path between the root term and these two leaf-terms we can find, not unexpectedly, the term *lyase activity*. Hence the graph confirms the expected dominant annotation with the term *lyase activity* and sibling terms in a protein Set that is itself a sub-set of proteins belonging to Polysaccharide Lyase class protein family of the CAZy classification. Hence, in our current PL1 Set example the *lyase activity* term node would be a good candidate for a *re-root* point. That is further supported by the p-values and IC-based term score statistics (respectively 2,006 × 10^−18^ and 0.171). Fig. 5 depicts the generated page footer containing the term names and respective statistics sorted by p-value. Given that the IC-based term score is the product of the IC of a term (in a given corpus) and its respective frequency in a given Set, it then provides a measure of *specific representativity* of a term in that Set. In other words, by having a high score *lyase activity* is one of the most frequent of the most specific annotation terms in the Set. However, since this is not a leaf-term there is a potential for annotation extension of the proteins not annotated beyond this term. We can then perform GRYFUN *re-root* operation on the *lyase activity* node which results in a new sub-graph as depicted in Fig. 6. Thus, in this case three separate sets of proteins (one for each of the two leaf-siblings in the current Set and another for all the proteins annotated to the *lyase activity* term) can be exported and submitted to annotation analysis (manual or otherwise) that could lead to annotation extension.

On the other hand, despite our terms deemed of interest and relevance being identified as enriched (statistically significant), the ranking of their p-values does not entirely match the *annotation flow*. However, we have to consider that the background against which the enrichment hypothesis was being tested was only the remainder of PL sets, which was therefore expected to retain a degree of functional closeness, that is, a number of these activities would also be present in other Sets within this Collection. Nevertheless, when we use all CAZy database families as the Collection (and hence background), the enrichment results are closer to the expected values. Table 1 displays a sample of the term enrichment list, ranked by p-value, of the PL1 family (set) relation to a background of 237 CAZy families of catalytic classes Glycoside Hydrolases (GH), GlycosylTransferases (GT), Polysaccaride Lyases and Carbohydrate Esterases (CE). The top ranked terms here match the annotation flow as depicted in Fig. 6, thus illustrating the importance of defining a good background if a reliable enrichment analysis is desired.

**Table 1:** Term enrichment p-values for the PL1 Set significant terms (alpha = 0.01) while using the complete CAZy Collection as background.

term name	p-value
lyase activity	< 5.315 × 10^−248^
pectate lyase activity	5.315 × 10^−248^
pectin lyase activity	2.558 × 10^−094^
metal ion binding	5.068 × 10^−056^
molecular_function	6.276 × 10^−008^
pectinesterase activity	1.146 × 10^−007^
catalytic activity	7.547 × 10^−004^
peroxidase activity	7.531 × 10^−003^

**Fig 7:**
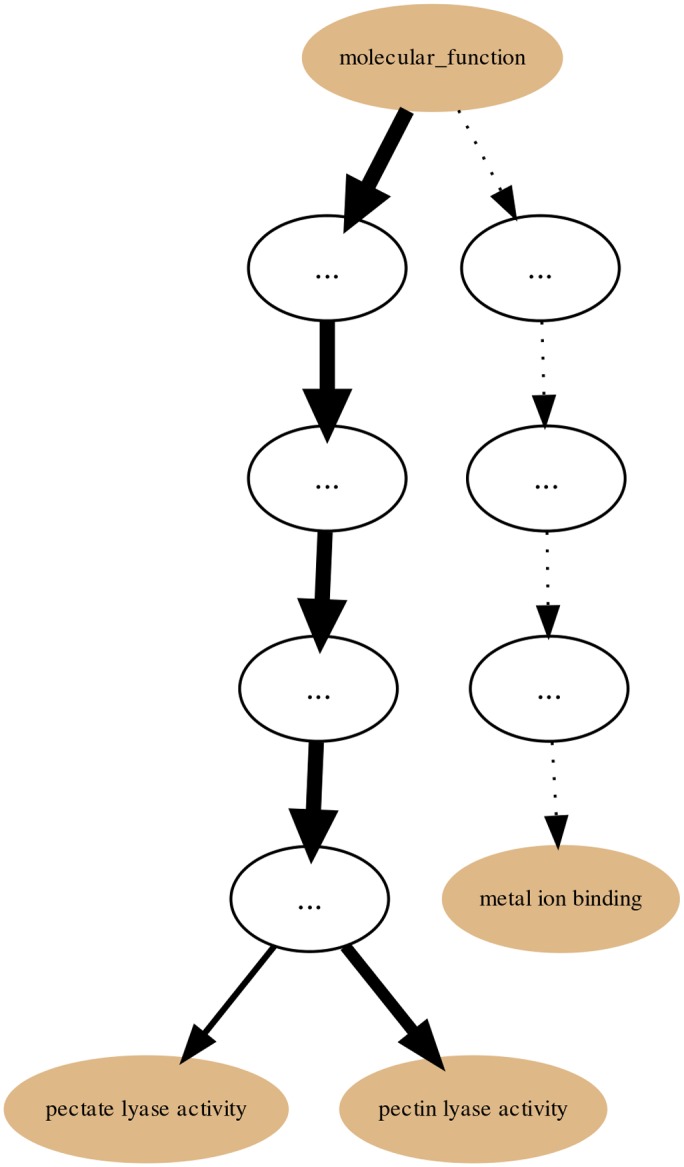
PL1 Set non-IEA annotation graph for the GO *molecular function* sub-ontology. Annotation graph subsuming the PL1 (within the CAZy Collection) Set GO *molecular function* sub-ontology annotations without electronic annotations (IEA).

In addition, we used the Evidence Code Filter to filter out Inferred Electronic Annotations (IEA) and generate a new annotation graph for the PL1 Set. The resulting graph seen in Fig. 7 is simpler than the one in Fig. 4 where all available annotations were used regardless of their Evidence Codes. Because the bulk of all annotations consist of IEA annotations the PL1 Set only has 32 out of 564 proteins with non-IEA annotations. Hence, this filtering focuses the PL1 Set on its annotations considered to be of higher quality but at the cost of coverage. Furthermore, the simplification of the graph also matches that of the previously shown term enrichment (using all annotations) thus reinforcing the previous enrichment results.

**Fig 8:**
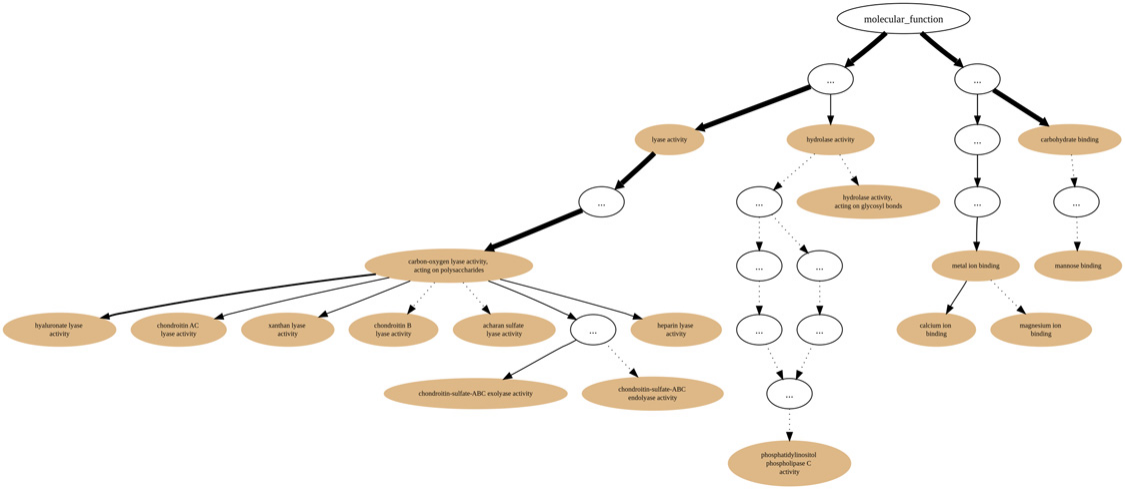
PL8 annotation graph. Annotation graph subsuming the PL8 (CAZy family) Set GO *molecular function* sub-ontology annotations.

Furthermore, we also generated the GO annotation graph (using all Evidence Codes) in the *molecular function* sub-ontology for the 197 proteins that amount to the PL8 Set (Family) with the CAZy Collection as background. Table 2 shows the term annotation occurrence numbers, IC-based scores and p-values for the enriched terms in Set PL8. The top three statistically significant terms are also the most representative in terms of Information Content as can be seen by the values of the IC-based score. Among these three, the term *carbon-oxygen lyase activity, acting on polysaccharides* has the higher score (0.426). When considering that score in conjunction with the “annotation flow” shown on Fig. 8, we see that about 70% of the proteins are not annotated beyond this term thus making it a good “pivot point” to attempt annotation extension. The remaining proteins that are annotated with terms that are its descendants are mostly (44 proteins) annotated to the term *hyaluronate lyase activity*, the third most IC significant term (IC-based score = 0.147). Furthermore, family PL8 has 3 sub-families (1–3) [21], which in our set are constituted by 88, 33, 4 proteins respectively. Additionally, there are still 77 remaining proteins in Set PL8 that are not classified into any of the sub-families. The examination of PL8 sub-family 1 shows that it is characterized by (27) proteins that are annotated to the *hyaluronate lyase activity* term (this term is enriched in the sub-family using the family as background). On the other hand, sub-families 2 and 3 are scarcely annotated beyond the term *carbon-oxygen lyase activity, acting on polysaccharides* and thus do not provide statistical support for what are their most specific representative activities. Hence, further annotation would be required for the members of sub-families 2 and 3 in order to assess a more specific functional profile for them.

**Table 2:** Term annotated occurrence (occ) number, IC-based term score and enrichment p-values for the PL8 Set significant terms (alpha = 0.01) while using the complete CAZy Collection as background.

term name	occ	IC-based score	p-value
carbon-oxygen lyase activity, acting on polysaccharides	196	0.426	< 2.531 × 10^−225^
carbohydrate binding	191	0.268	2.531 × 10^−225^
hyaluronate lyase activity	44	0.147	2.081 × 10^−124^
chondroitin AC lyase activity	3	0.012	3.996 × 10^−009^
xanthan lyase activity	2	0.010	2.531 × 10^−006^
chondroitin-sulfate-ABC exolyase activity	2	0.009	2.531 × 10^−006^
heparin lyase activity	2	0.008	7.042 × 10^−005^
chondroitin-sulfate-ABC endolyase activity	1	0.004	1.595 × 10^−003^
acharan sulfate lyase activity	1	0.005	1.595 × 10^−003^
chondroitin B lyase activity	1	0.005	3.187 × 10^−003^
phosphatidylinositol phospholipase C activity	1	0.003	6.365 × 10^−003^
metal ion binding	7	0.004	9.978 × 10^−003^

### YHTP2008

**Fig 9:**
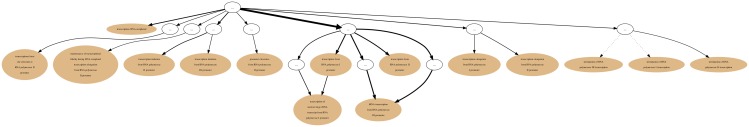
Complex 3 Set annotation graph *re-rooted* at the *RNA biosynthetic process* term. Annotation graph of the Complex 3 (YHTP2008 Collection) Set for the GO *biological process* sub-ontology *re-rooted* at the *RNA biosynthetic process* term.

We have chosen to generate the *biological process* sub-ontology annotation graph for Complex 3 of our Collection containing 133 YHTP2008 complexes we had previously loaded into GRYFUN. This complex had been reported to contain multiple complexes, sharing sub-units extensively, specifically all three RNA polymerases [22]. Table 3 shows the terms found enriched in this Complex (for a significance level of 0.01). As expected, several terms related to RNA polymerase I-III processes can be found in the list of enriched terms. Supplementary S1 Fig. shows the respective annotation graph for Complex 3. Typically terms on the *biological process* sub-ontology are more interconnected (as opposed to the *molecular function* and *cellular component* sub-ontologies) and navigation can be more complicated. In this particular case, using the enrichment results alone does not clarify which node(s) to use for *re-rooting*, and hence simplify the graph. However, following the “annotation flow” we reach the term *RNA biosynthetic process* that annotates 37 of the 40 proteins in Complex 3 and is a parent to a number of enriched terms in this Set and thus a likely candidate for a *re-rooting* operation. The resulting annotation graph can be seen in Fig. 9 which is considerably easier to navigate than the original full annotation graph while retaining 11 of the 19 terms originally found enriched. This sub-set of 11 terms also present in the re-rooted graph also captures 3 of the most specific and representative terms for this protein Set (as indicated by their IC-based term score): *transcription from RNA polymerase I promoter* (0.229), *transcription from RNA polymerase II promoter* (0.120) and *tRNA transcription from RNA polymerase III promoter* (0.345).

**Table 3:** Term enrichment p-values for Complex 3 Set significant terms (alpha = 0.01) for the YHTP2008 Collection.

term name	p-value
mRNA metabolic process	< 7.40 × 10^−028^
DNA metabolic process	< 7.40 × 10^−028^
tRNA transcription from RNA polymerase III promoter	7.404 × 10^−028^
transcription, RNA-templated	7.927 × 10^−018^
transcription from RNA polymerase I promoter	4.223 × 10^−012^
transcription from RNA polymerase II promoter	5.719 × 10^−012^
transcription of nuclear large rRNA transcript from RNA polymerase I promoter	6.694 × 10^−010^
transcription initiation from RNA polymerase II promoter	5.855 × 10^−009^
promoter clearance from RNA polymerase II promoter	3.006 × 10^−005^
transcription initiation from RNA polymerase III promoter	3.006 × 10^−005^
ribosome biogenesis	9.137 × 10^−005^
transcriptional start site selection at RNA polymerase II promoter	1.176 × 10^−004^
transcription elongation from RNA polymerase I promoter	5.622 × 10^−04^
phosphate-containing compound metabolic process	9.432 × 10^−004^
maintenance of transcriptional fidelity during DNA-templated …	
…transcription elongation from RNA polymerase II promoter	9.913 × 10^−004^
negative regulation of transcription elongation from RNA polymerase I promoter	9.913 × 10^−004^
regulation of rRNA processing	2.914 × 10^−003^
positive regulation of translational initiation	2.914 × 10^−003^
7-methylguanosine mRNA capping	5.710 × 10^−003^

Additionally, we submitted the Cluster 3 (and respective background) proteins to the GOrilla enrichment analysis and visualization tool. Supplementary S2 Fig. shows the respective annotation graph generated by GOrilla. The graph produced by GOrilla is less extensive (in number of nodes) than the one generated by GRYFUN. That can easily be explained by the different annotation corpus used by the two tools. Although the graph generated by GRYFUN is originally more extensive, that can be dynamically changed by using the *re-rooting* feature and thereby “reducing” the graph to its more relevant sub-graph branches. Interestingly, GOrilla graphs represent the statistical significance found for each term by coloring the respective nodes according to a p-value color scale, while GRYFUN uses proportional edge thickness to represent the previously described “annotation flow”. Regarding the enriched terms, GOrilla detected 45 terms for a less restrictive p-value cut-off of 1.0 × 10^−3^ (see Supplementary S1 File). On the other, for the same cut-off, GRYFUN only identifies 16 terms as significant (see Supplementary S2 File) of which 12 are contained in the GOrilla list. The difference in number of enriched terms arises from the use of the Elim procedure [16, 17] in GRYFUN. As previously explained, this technique mitigates the statistical dependencies between nodes downplaying ancestor nodes. This is a desired effect, since (for a similar level of annotation quality) a more specific annotation is preferable to a general annotation. Through the inspection of the GOrilla graph it can be seen that the bulk of the terms it identifies as significant are ancestors of the terms *RNA biosynthesis process* and *transcription DNA-templated*. On the other hand, through GRYFUN we can see that both these terms annotate 37 of the 40 proteins in the Set. Furthermore, 10 out of the 12 terms on the intersection of the enrichment lists from GRYFUN and GOrilla are children of these two terms. Therefore, from an annotation perspective the ancestors of these two terms are less interesting and GRYFUN becomes advantageous by reducing their relevance through the internal use of the Elim technique.

### MEROPS

**Fig 10:**
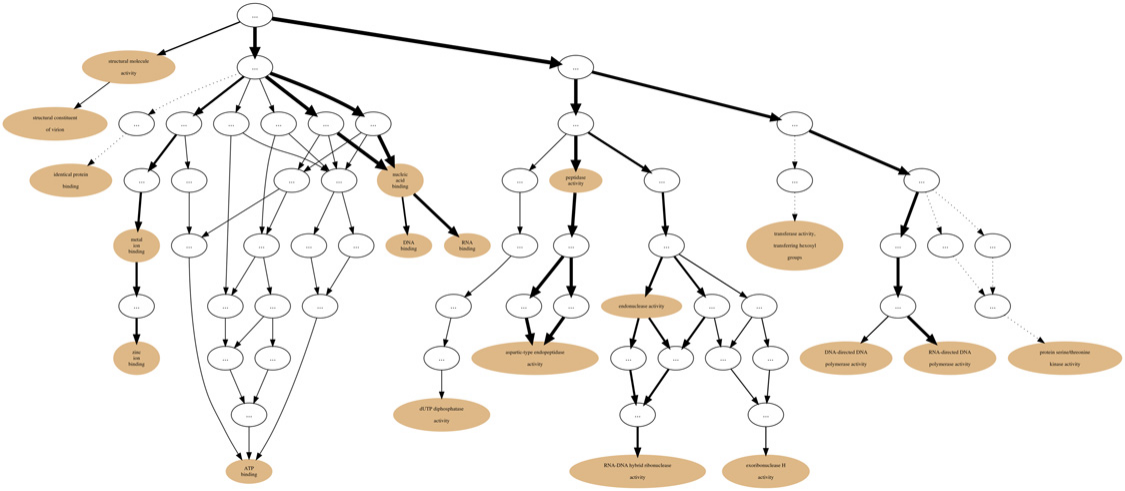
A2 annotation graph. Annotation graph subsuming the A2 Set (MEROPS Collection) GO *molecular function* sub-ontology annotations.

Within the MEROPS families previously imported into a GRYFUN Collection we randomly picked Set (family) A2 and generated its annotation graph and associated statistics for the GO *molecular function* sub-ontology. The annotation graph is shown in Fig. 10 while Table 4 displays the associated statistics. The MEROPS website (http://merops.sanger.ac.uk/cgi-bin/famsum?family=A2) describes peptidase family A2 as containing “endopeptidases with catalytic sites of aspartic type”. Table 4 shows that the enriched term *aspartic-type endopeptidase activity* is the most specific and prevalent one (IC-based term score = 0.222; annotates 77% of the Set), thus supporting the MEROPS family classification for this Set. Additional high-scoring terms in this Set are *RNA-directed DNA polymerase activity*, *RNA-DNA hybrid ribonuclease activity* and *RNA binding* all of which are functions inherently related to the reported family type, HIV-1 retropepsin. Furthermore, for this family the annotation graph is easy to navigate and there are several “annotation flow” paths flowing towards specific relevant terms. Hence, there are still potential annotation extension opportunities down each of these paths since none of the significant terms annotates all the proteins in the dataset.

**Table 4:** Term annotated occurrence (occ) number, IC-based term score and enrichment p-values for the A2 Set significant terms (alpha = 0.01) while using the complete collection of MEROPS Collection as background.

term name	occ	IC-based term score	p-value
RNA-directed DNA polymerase activity	109	0.194	7.24 × 10^−271^
RNA-DNA hybrid ribonuclease activity	68	0.158	5.01 × 10^−171^
RNA binding	105	0.106	3.27 × 10^−165^
aspartic-type endopeptidase activity	120	0.222	1.05 × 10^−131^
exoribonuclease H activity	16	0.077	4.80 × 10^−045^
nucleic acid binding	134	0.074	1.82 × 10^−041^
DNA binding	48	0.035	4.86 × 10^−035^
DNA-directed DNA polymerase activity	18	0.034	5.18 × 10^−032^
zinc ion binding	73	0.091	3.36 × 10^−031^
structural molecule activity	33	0.039	3.86 × 10^−022^
structural constituent of virion	5	0.013	1.70 × 10^−014^
dUTP diphosphatase activity	4	0.012	4.88 × 10^−011^
phosphotransferase activity, alcohol group as acceptor	1	0.001	3.57 × 10^−003^
transferase activity, transferring hexosyl groups	1	0.002	3.57 × 10^−003^

### Gene Expression Micro-array Data

Performance of GRYFUN was compared with that of two publicly available platforms for GO term enrichment (GOrilla & DAVID), with biological process terms being investigated, for a list of upregulated genes taken from a CF micro-array study [25]. GRYFUN identified 90 GO terms (out of 2006) considered statistically significant (alpha = 0.01) annotating the 150 protein identifiers submitted, compared to the 5 identified by DAVID at the same significance level, and the 56 identified by GOrilla (default alpha = 0.001). Some terms were among the most significant identified by all three platforms, while others were only considered significant by one or two platforms, and there were some variations in the number of genes identified as annotated under specific GO terms (see Table 5). The variations in the number of annotation occurrences for each term stem from the fact that each of the enrichment tools does not rely exactly on the same releases of annotation databases. GRYFUN also identified as being enriched several GO terms of definite biological significance in the pathophysiology of CF (eg, *Positive Regulation of Cell Differentiation*, *Programmed Cell Death*) which were undetected by other platforms (see Table 5).

**Table 5:** Comparison of GO term enrichment analyses of micro-array data by GRYFUN, DAVID and GOrilla. Selected examples of GO terms found to be enriched in list of differentially expressed genes (upregulated in cystic fibrosis nasal epithelium [25]) by GRYFUN, DAVID or GOrilla. Occurrence (occ) numbers and p-values are shown. “Not found” means the GO term was not considered significant.

Terms (biological process)	GRYFUN		DAVID		GOrilla	
	p-value	occ	p-value	occ	p-value	occ
Response to Wounding	3.6 × 10^−08^	12	3.2 × 10^−03^	13	2.1 × 10^−04^	11
Immune Response	2.2 × 10^−05^	15	4.0 × 10^−03^	15	8.8 × 10^−05^	21
RNA Biosynthetic Process	< 1.0 × 10^−13^	15	Not found		Not found	
Programmed Cell Death	< 1.0 × 10^−13^	11	Not found		Not found	
Positive Regulation of Cell Differentiation	2.7 × 10^−06^	14	Not found		3.0 × 10^−06^	20
Negative Regulation of Cell Communication	Not found		7.5 × 10^−03^	8	9.6 × 10^−04^	19
Inflammatory Response	Not found		9.6 × 10^−03^	9	3.1 × 10^−04^	11
Ectoderm Development	Not found		9.6 × 10^−03^	7	Not found	

The relevance of enriched processes to CF, or any other condition being studied, has for GRYFUN as for other enrichment platforms including GOrilla and DAVID, to be assessed by the user, based on knowledge of the processes involved. Additionally, when analysing micro-array data such as this, where there is a high number of biological processes involved, the use of the occurrence number (on statistically enriched terms) can be a quick indicator of how general a process might be. Among the 90 significant GO terms identified by GRYFUN in our CF data set, approximately 30 had occurrence numbers between 5 and 15, and represented the most functionally relevant, including some of those shown in Table 5. The identification of significantly enriched processes by DAVID and not by GRYFUN or GOrilla, and vice versa, may result from the different backgrounds used: the default DAVID background is composed of all human genes with at least one annotation in the category being analysed, whereas the GRYFUN/GOrilla backgrounds are user-defined, in this case being composed of genes represented on the micro-array for which a UniProt accession number or Gene Symbol (respectively) was available. Future implementation of a default genome-wide background might standardize the results of enrichment analyses, but the greater number of significant terms produced by GRYFUN in the present analysis could nevertheless prove useful in generating functional hypotheses. For example, the three GO terms identified by GRYFUN in Table 5, of which only one was found by GOrilla and none by DAVID (*RNA Biosynthetic Process*, *Programmed Cell Death*, *Positive Regulation of Cell Differentiation*), all have important roles in CF-mediated airway pathology [27–29].

The dataset generated here is subsumed by over two thousand (highly interconnected) GO terms (in the biological function sub-ontology), that in turn, renders an extremely complex interactive graph of difficult navigation and interpretation. This is a limitation of our current graph rendering engine. However, GRYFUN provides the possibility to download the underlying graph file (by pressing the button “Export.dot” on the Set header) which can then be opened with a suitable external viewer application such as the free and cross-platform ZGRViewer (http://zvtm.sourceforge.net/zgrviewer.html). In the future, we plan to implement strategies that help deal with very big graphs, such as pre-rendering additional filters and partial iterative graph loading. Notwithstanding, when a graph of difficult interpretation (due to number of nodes and edges) is generated it is currently possible to immediately perform *re-rooting* operations from the associated term table while guided by the presented statistics. These *re-rooting* operations will then result in smaller and more interpretable branches of the original graph.

### Future Work

In the future we expect to extend the number of identifiers allowed and handled as input by GRYFUN, with the possibility of uploading customised annotation mapping files. Additionally, we intend to add features that enable the direct handling of data (such as directly creating new sets out of sub-selections of a set) in the main Graph Exploring interface page and supply pre-loaded Collections as *background* options. Regarding the graph output we plan to implement additional rendering options and apply other strategies in order to more efficiently deal with especially larger graphs. More importantly, we will be adding further metrics, that we are currently developing, to measure and perform global assessments of the coherence and cohesiveness of functional annotations and overall completeness of annotation in a protein set. Furthermore, all code is available (under an MIT license) on a public GIT server (https://bitbucket.org/hpbastos/gryfunserver/) so that anyone can modify, contribute to or simply deploy GRYFUN in their own servers.

## Supporting Information

S1 Fig Complex 3 Set annotation graph generated by GRYFUN. Annotation graph of the Complex 3 (YHTP2008 Collection) Set for the GO *biological process* sub-ontology graph generated by GRYFUN.
(TIF)

S2 Fig Complex 3 Set enrichment graph generated by GOrilla. Enrichment graph of the Complex 3 (YHTP2008 Collection) Set for the GO *biological process* sub-ontology generated by GOrilla.
(TIF)

S1 File Complex 3 Set enrichment results spreadsheet file output by GOrilla. Enrichment results spreadsheet file of the Complex 3 (YHTP2008 Collection) Set for the GO *biological process* sub-ontology generated by GOrilla.
(XLS)

S2 File Complex 3 Set enrichment results TSV file output by GRYFUN. Enrichment results TSV file of the Complex 3 (YHTP2008 Collection) Set for the GO *biological process* sub-ontology generated by GRYFUN.
(TSV)
