# Supplementary figures and images for: GRYFUN: A Web Application for GO Term Annotation Visualization and Analysis in Protein Sets
Source: PLoS One. 2015 Mar 20;10(3):e0119631. doi: 10.1371/journal.pone.0119631 (PMC4368792; doi:10.1371/journal.pone.0119631)

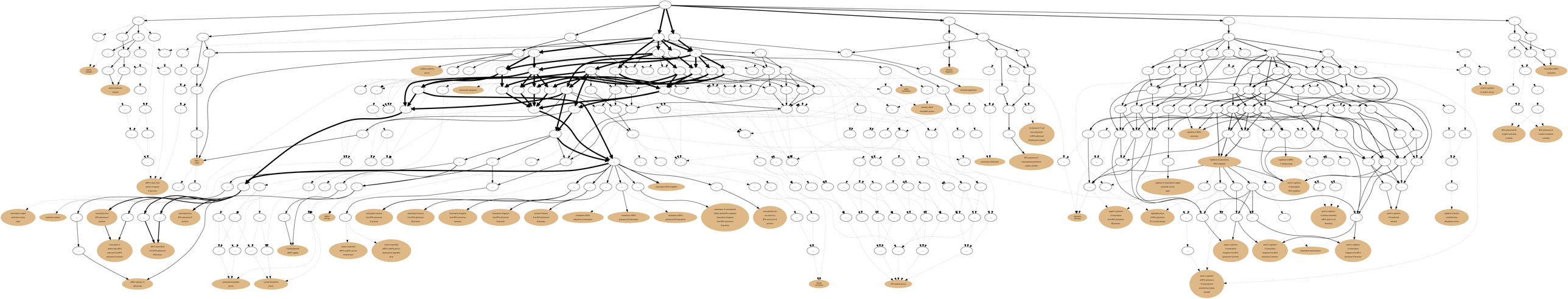

Supplement: S1 Fig — Annotation graph of the Complex 3 (YHTP2008 Collection) Set for the GO biological process sub-ontology graph generated by GRYFUN. (TIF) [file pone.0119631.s001.tif]

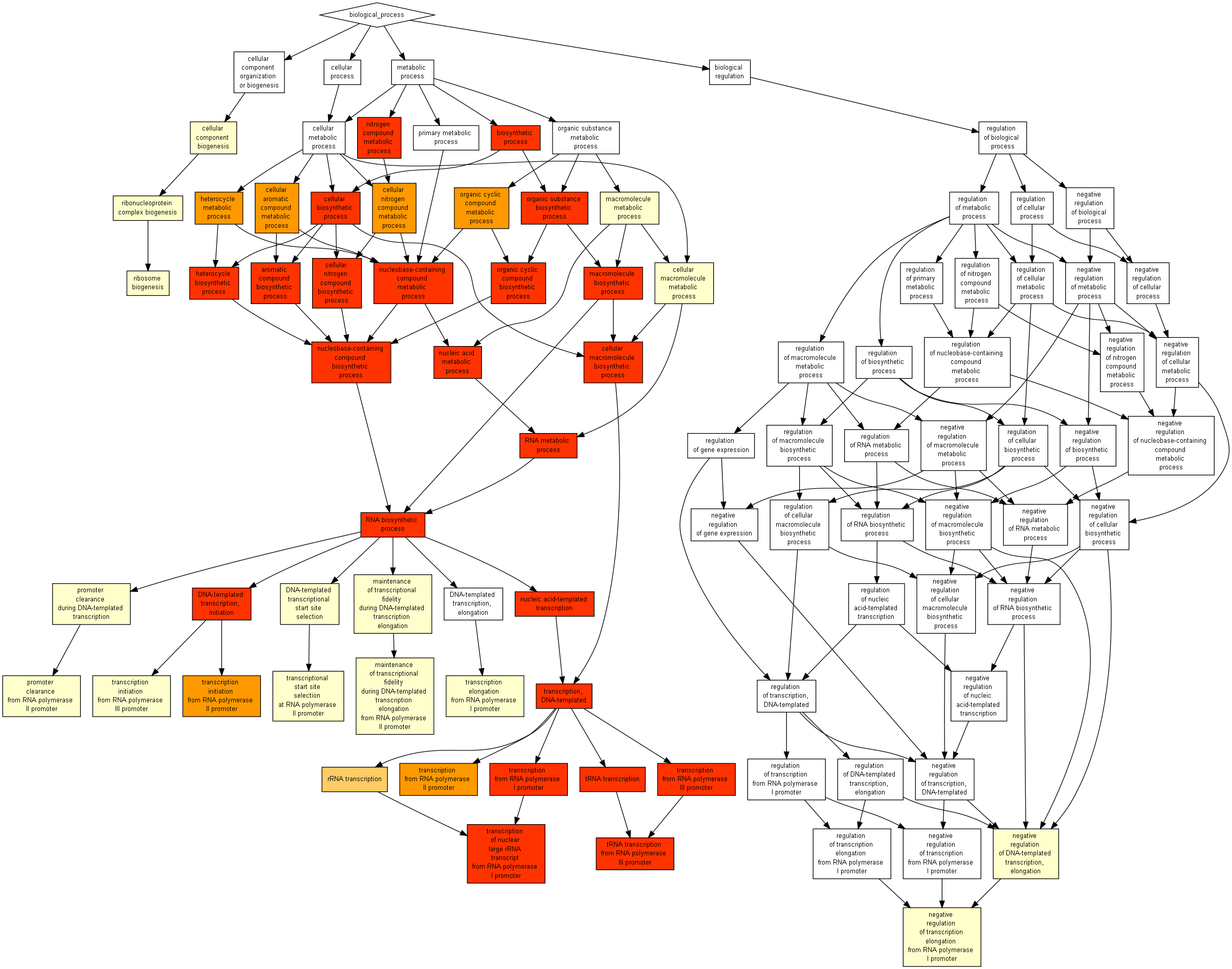

Supplement: S2 Fig — Enrichment graph of the Complex 3 (YHTP2008 Collection) Set for the GO biological process sub-ontology generated by GOrilla. (TIF) [file pone.0119631.s002.tif]
